# Cyclin D1 unbalances the redox status controlling cell adhesion, migration, and drug resistance in myeloma cells

**DOI:** 10.18632/oncotarget.9901

**Published:** 2016-06-07

**Authors:** Sophie Bustany, Jérôme Bourgeais, Guergana Tchakarska, Simon Body, Olivier Hérault, Fabrice Gouilleux, Brigitte Sola

**Affiliations:** ^1^ Université de Caen Normandie, EA4652 (MILPAT), MICAH Team, Caen, France; ^2^ Université François Rabelais, CNRS UMR 7292 (GICC), LNox Team, Tours, France; ^3^ Service d'Hématologie Biologique, CHRU Tours, Tours, France; ^4^ Present address: Cytogenetics Laboratory, Research Institute, McGill University Health Centre, Montréal, Canada

**Keywords:** reactive oxygen species, p44/42 mitogen-activated protein kinase, pomalidomide, carfilzomib, NADPH oxidase

## Abstract

The interactions of multiple myeloma (MM) cells with their microenvironment are crucial for pathogenesis. MM cells could interact differentially with their microenvironment depending on the type of cyclin D they express. We established several clones that constitutively express cyclin D1 from the parental RPMI8226 MM cell line and analyzed the impact of cyclin D1 expression on cell behavior. We performed a gene expression profiling study on cyclin D1-expressing *vs*. control cells and validated the results by semi-quantitative RT-PCR. The expression of cyclin D1 altered the transcription of genes that control adhesion and migration. We confirmed that cyclin D1 increases cell adhesion to stromal cells and fibronectin, stabilizes F-actin fibers, and enhances chemotaxis and inflammatory chemokine secretion. Both control and cyclin D1-expressing cells were more resistant to acute carfilzomib treatment when cultured on stromal cells than in suspension. However, this resistance was specifically reduced in cyclin D1-expressing cells after pomalidomide pre-treatment that modifies tumor cell/microenvironment interactions. Transcriptomic analysis revealed that cyclin D1 expression was also associated with changes in the expression of genes controlling metabolism. We also found that cyclin D1 expression disrupted the redox balance by producing reactive oxygen species. The resulting oxidative stress activated the p44/42 mitogen-activated protein kinase (or ERK1/2) signaling pathway, increased cell adhesion to fibronectin or stromal cells, and controlled drug sensitivity.

Our results have uncovered a new function for cyclin D1 in the control of redox metabolism and interactions of cyclin D1-expressing MM cells with their bone marrow microenvironment.

## INTRODUCTION

Multiple myeloma (MM) remains an incurable hemopathy characterized by the accumulation of clonal plasma cells within the bone marrow and the overproduction of monoclonal immunoglobulin. The clinical signs of MM are immunodeficiency, recurrent infections, renal failure, and bone lesions [1]. Myeloma tumor cells are characterized by genomic abnormalities including chromosomal number and structural variations. Almost all MM tumors express one of the three cyclin D proteins. Thus, it has been proposed that dysregulation of one *CCND* gene encoding cyclin D is a unifying event of MM pathogenesis [2]. Paradoxically, MM tumor cells are mostly low proliferating even though the major role of cyclins D is to regulate the progression through the G1 phase and the G1 to S phase transition of the cell cycle [3].

The oncogenic potential of cyclin D1, is largely due to its cell cycle regulating function when associated with its cyclin-dependent kinase (CDK)4/6 partners [4]. In addition, other CDK-dependent or -independent non-canonical roles of cyclin D1 may be important for tumor initiation, maintenance, progression, and metastasis [5]. Almost 45% of tumors in MM patients express cyclin D1 but, paradoxically, this expression is associated with a favorable prognosis [6]. We established two series of clones derived from RPMI8226 MM cells expressing either a cyclin D1-green fluorescent (GFP) fusion protein (D1-GFP) or GFP alone to elucidate the molecular functions of cyclin D1 in MM [7]. We found that cyclin D1 alters the expression of genes involved in the regulation of the cell cycle, cell proliferation, apoptosis, and protein synthesis, in agreement with the well-known functions of cyclin D1 but, unexpectedly, also of cell metabolism, including the redox balance. We further studied how cyclin D1 controls the redox status and how this affects cell adhesion, migration, and the response to drugs, in particular, cell adhesion-mediated drug resistance (CAM-DR).

## RESULTS

### Cyclin D1 expression in myeloma cells alters various cell functions

We previously established several clones expressing either GFP or cyclin D1(D1)-GFP fusion proteins from the RPMI8226 parental MM cells (hereafter referred to as 8226 cells) [7]. Two independent clones from each series were further used in this study. We verified the expression of the exogenous proteins both by flow cytometry and western blot analysis (Supplementary Figure 1A). As expected, D1-GFP-expressing clones proliferated more rapidly than GFP-expressing clones (Supplementary Figure 1B). This indicates that cyclin D1 was fully functional. We performed whole-genome expression profiling to identify genes for which the expression is altered by cyclin D1. As reported earlier [7], the comparison of GFP- and D1-GFP-expressing cells showed that cyclin D1 altered the transcription of genes involved in DNA and protein synthesis, cell cycle regulation, apoptosis, and inflammation as expected, but also genes involved in metabolism, membrane trafficking, and adhesion/migration [Gene Expression Omnibus: GSE59673].

### Cyclin D1 increases cell adhesion and migration, and chemokine secretion

**Figure 1:**
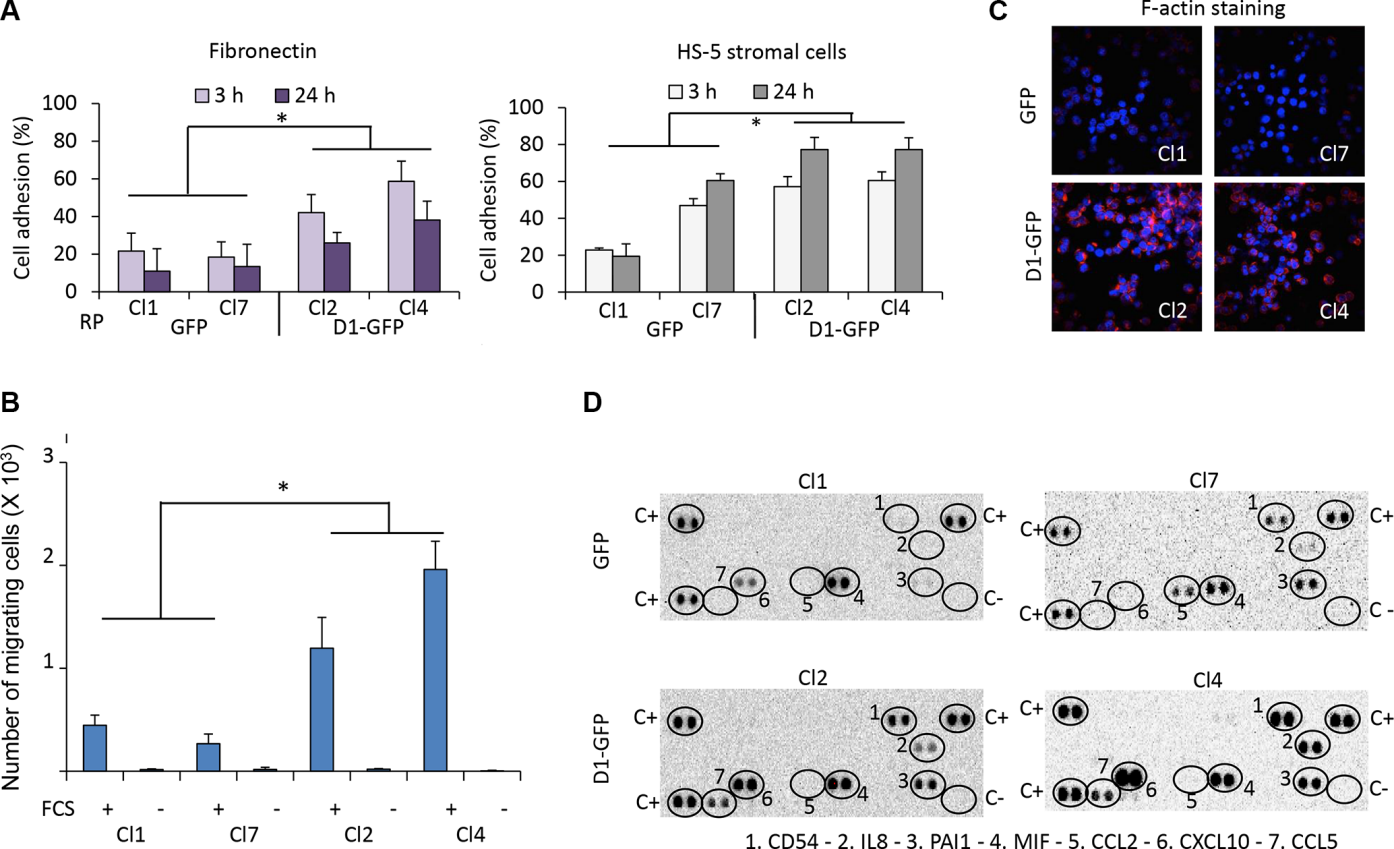
Cyclin D1 controls cell adhesion, cell migration, and cytokine production (**A**) 96-well plates were coated with fibronectin or HS-5 stromal cells. GFP- and D1-GFP-expressing clones were stained with calcein-AM and seeded. After incubation for 3 or 24 h at 37°C, non-adherent cells were removed by extensive washing. The plates were read with the Victor ×4 plate-reader. The percentage of cell adhesion was calculated by the ratio fluorescence of adherent cells/fluorescence of total cells x 100. Presented results corresponded to the mean of four independent experiments with five replicates. (**B**) GFP- and D1-GFP expressing clones were seeded in the top chamber of transwell inserts. The inserts were then placed in culture medium with FCS (+) or without FCS (−) as a control for specificity. The cells were incubated for 4 h at 37°C, and the number of migrating cells within the bottom of the insert was counted by flow cytometry. The results presented correspond to the mean of three independent experiments performed in triplicate. (**C**) GFP- and D1-GFP-expressing clones were cytospun on glass slides, stained with rhodamine-stained phalloidin for visualizing F-actin and counterstained with DAPI. The slides were analyzed with a confocal microscope (×180, magnification). (**D**) The Cytokine Array kit (panel A) was used for the detection of cytokines secreted in the culture medium by GFP- and D1-GFP clones. The assay procedure was performed according to the manufacturer's instructions. Spots corresponding to positive controls (C+), negative controls (C−), and produced cytokines are circled. *p < 0.05 with the *t*-test.

Cyclin D1 is involved in the regulation of adhesion and migration. Ablation of *CCND1* reduces migration of macrophages, fibroblasts, and mammary epithelial cells [8–11]. In breast cancer cells, cyclin D1 interacts with cytoskeletal proteins and controls migration [12]. In keratinocytes, cytoplasmic cyclin D1 regulates cell-matrix adhesion [13]. We assessed the capacity of GFP- and D1-GFP-expressing clones to adhere to fibronectin or HS-5 stromal cells after their staining with calcein-AM. Cyclin D1 expression increased cell adhesion to both substrates (Figure 1A). We assayed the migration capacity of the same clones using a chemotaxis assay in which cells seeded in transwell inserts are attracted by growth factors present in fetal calf serum (FCS). Cyclin D1 expression increased the migration capacity of cells (Figure 1B) which was confirmed by rhodamine-phalloidin staining of filamentous (F-) actin and confocal microscopy analysis (Figure 1C). We also observed increased adhesion and migration for other clones derived from LP1 and L363 parental MM cell lines expressing exogenous cyclin D1 (data not shown).

We used the Proteome Profiler™ Human Cytokine Array to detect which chemokines/cytokines were produced by cyclin D1-expressing cells. Cyclin D1 increased the production of CD54 or ICAM1, interleukin (IL)8, and CXCL10 (chemokine (C-X-C motif) ligand 10) also known as interferon γ-induced protein 10 (IP10). It also stimulated the production of CCL5 (chemokine (C-C) motif 5), also known as RANTES (regulated and normal T-cell expressed and secreted). These chemokines/cytokines are all involved in inflammatory processes and cell adhesion (Figure 1D).

We conclude that cyclin D1: 1) increases cell adhesion on fibronectin and stromal cells; 2) increases the synthesis of the adhesion molecule ICAM1; 3) increases the production of inflammatory chemokines such as IL8, IP10, and RANTES; and 4) favors cell migration.

### Pomalidomide decreases the cell adhesion-mediated drug resistance of cyclin D1-expressing myeloma cells

**Figure 2:**
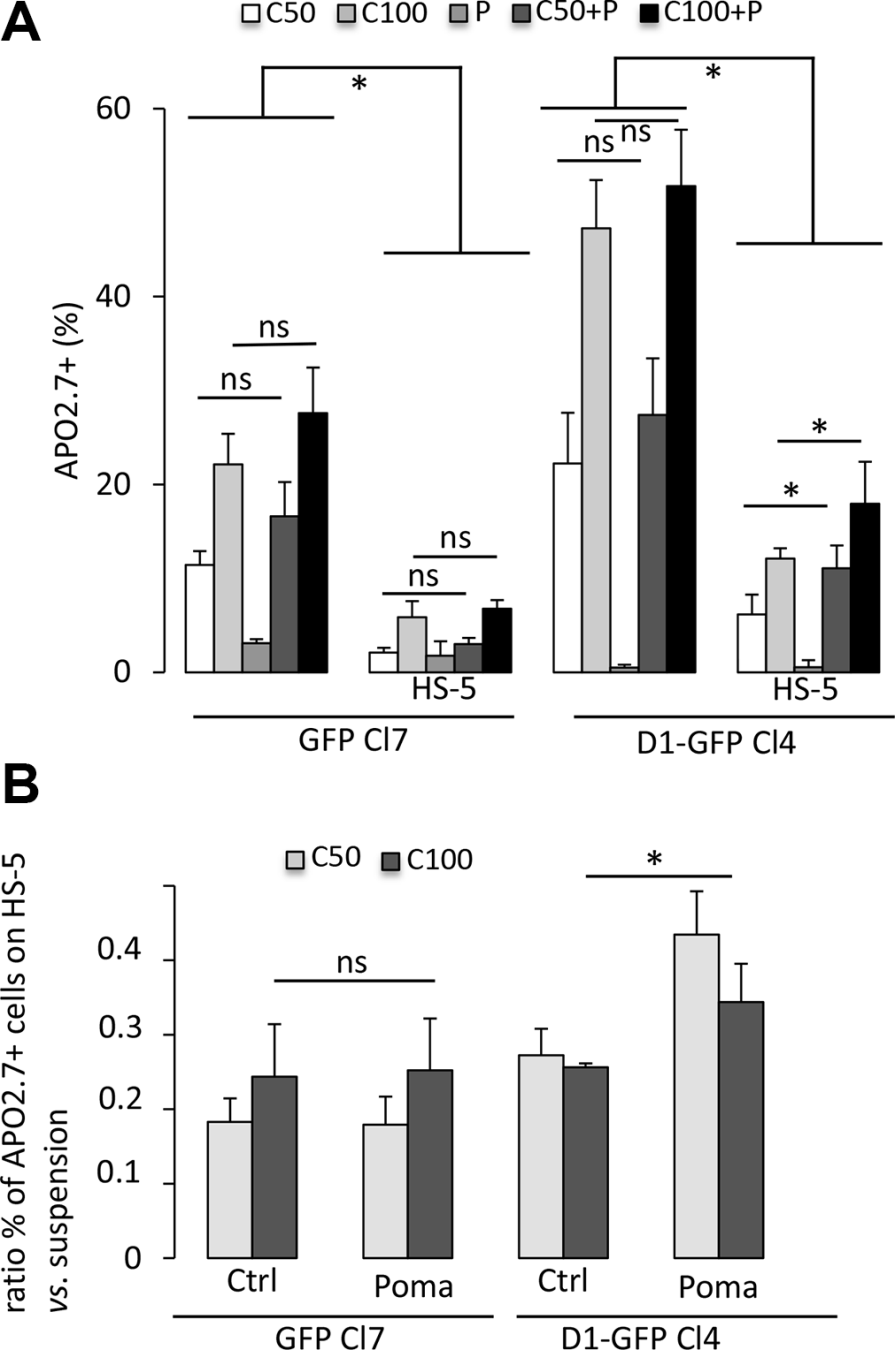
Cyclin D1 controls the sensitivity of cells to carfilzomib (**A**) GFP-expressing Cl7 and D1-GFP-expressing Cl4, either in suspension or in co-cultures with HS-5 cells, were treated with 1 μM pomalidomide (P) or with vehicle, as a control, for 72 h. The cells were then treated with 50 nM (C50) or 100 nM (C100) carfilzomib for 1 h and further cultured for 24 h. The cells were then stained with anti-APO2.7 Ab and analyzed by flow cytometry. At least, 2 × 10^4^ events were gated for each culture condition. The mean of four independent experiments is indicated in the graph together with the SD. (**B**) CAM-DR was calculated by the formula: percentage of APO2.7-positive cells in co-culture on HS-5 cells/percentage of APO2.7-positive cells in suspension. The mean ± SD of four independent experiments is indicated in the graph. *p < 0.05 with the *t*-test.

Carfilzomib is a second-generation proteasome inhibitor for the treatment of MM [14]. However, its *in vivo* half-life is very short [15], similar to that of bortezomib, widely used in clinical practice [16]. We either chronically (5–30 nM for 24 h) or acutely (50–500 nM for a 1 h-treatment followed by a 24 h-culture) administered carfilzomib or bortezomib to cultured GFP- and D1-GFP-expressing clones and analyzed cell viability using an MTS-based assay. D1-GFP-expressing cells were more sensitive to bortezomib than GFP-expressing to chronic treatment with bortezomib but not carfilzomib (Supplementary Figure 2A). However, cyclin D1 increased the sensitivity to acute treatments with either bortezomib or carfilzomib (Supplementary Figure 2B). We further confirmed this result by quantifying apoptotic (APO2.7-positive) cells after carfilzomib treatment of cells cultured either in suspension or on a layer of HS-5 stromal cells (Figure 2A). The interaction MM with bone marrow cells is responsible for CAM-DR [17–21]. Consistent with these observations, both GFP- and D1-GFP-expressing cells cultured on HS-5 cells were more resistant to carfilzomib than when cultured in suspension (Figure 2A). The co-culture of 8226-, LP1, and L363-derived clones on fibronectin-coated plates demonstrated CAM-DR for all clones (data not shown). Immunomodulators (IMIDs), such as pomalidomide are drugs that modify the interactions between tumor cells and their microenvironment by targeting adhesion proteins [22]. GFP- and D1-GFP-expressing clones, cultured in suspension or on stromal cells, were co-treated with pomalidomide and carfilzomib and the induced apoptotic response evaluated. Treatment with 1 μM pomalidomide for 72 h did not trigger apoptotic death of cells cultured in either setting (Figure 2A). The combination of carfilzomib and pomalidomide did not have an additive effect on GFP-expressing cells. In contrast, co-treatment increased the apoptotic response of cyclin D1-expressing cells when cultured on HS-5 cells (Figure 2A), and significantly decreased CAM-DR (Figure 2B), thus uncovering a cell-adhesion dependent effect of cyclin D1 on drug resistance. Taken together, these observations suggest that cyclin D1 decreases the sensitivity of MM cells to carfilzomib by modulating of MM cells/microenvironment interactions.

### Cyclin D1 generates reactive oxygen species and perturbs the redox balance

**Figure 3:**
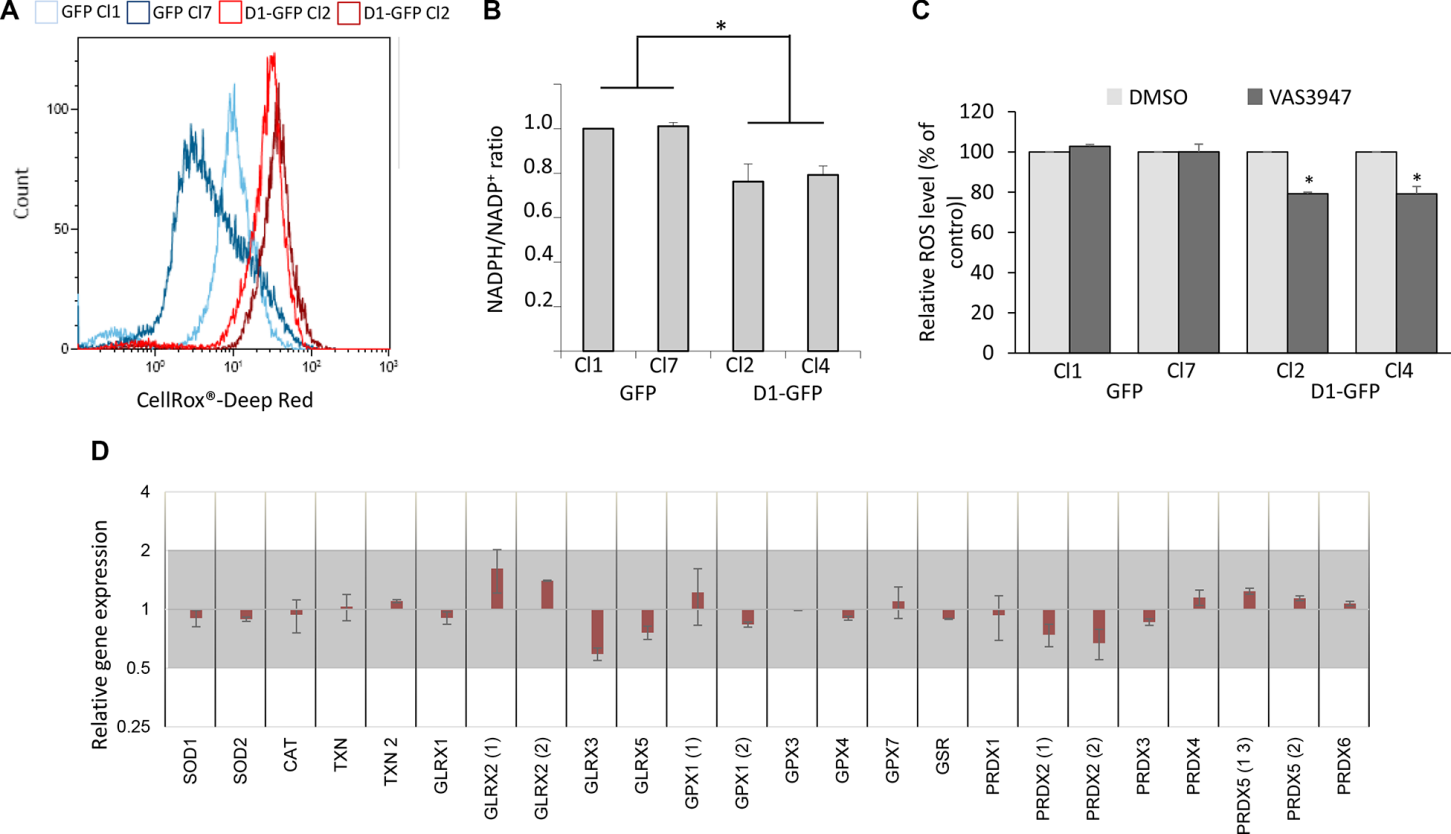
Cyclin D1 modifies the cell redox balance (**A**) GFP-expressing cells (Cl1 and Cl7), and D1-GFP-expressing cells (Cl2 and Cl4) (10^5^ cells) were incubated with the ROS sensitive fluorescent probe CellROX Deep Red and intracellular ROS levels were determined by flow cytometry. At least 2 × 10^4^ events were gated for each clone. (**B**) NADP and NADPH pools were extracted from 10^6^ cells and quantified by spectrometry. The NADP/NADPH ratio was calculated. Means ± SD from three independent experiments are presented. (**C**) GFP- (Cl1 and Cl7), and D1-GFP-expressing cells (Cl2 and Cl4) were treated with 1 mM NAC overnight or EtOH (Ctrl) and stained with CellROX to detect relative ROS levels (% of control) in GFP+ cells by flow cytometry as already described. At least 2 × 104 events were gated for each clone. The experiment was performed four times; the means ± SEM are shown on the graph. *p < 0.05, **p <0.01 by the *t*-test. (**D**) The level of antioxidant gene expression was compared between GFP Cl1/Cl7 and D1-GFP Cl4/Cl2 by a semi-quantitative RT-PCR. The results are presented as the fold change (2^−ΔΔCt^ values) of D1-GFP expressing cells *vs*. GFP-expressing cells relative to the internal control (*GAPDH* gene) normalized to 1. Mean values ranging from 0.5 to 2 (grey area) are not considered to be significant (n = 3 in triplicate, data are expressed as the mean ± SD). *SOD3, GPX2, GPX5*, and *GPX6* genes were not expressed.

Reactive oxygen species (ROS) are essential mediators of cell adhesion and migration [23]. We thus analyzed the redox state of cyclin D1-expressing clones. We used the CellROX^®^ Deep Red Reagent, a fluorogenic probe, to measure the cellular oxidative status. Cyclin D1 increased the production of ROS (Figure 3A). ROS are mainly produced by mitochondria and a family of NADPH oxidases or NOX [24]. We and others have shown that cyclin D1 can negatively regulate mitochondrial respiration [25, 26]. We further analyzed the metabolic profiles of GFP- and D1-GFP-expressing cells using the Seahorse XF96 analyzer which simultaneously records mitochondrial respiration and glycolysis. D1-GFP-expressing cells had the same oxygen consumption rate (OCR) as GFP-expressing cells (Supplementary Figure 3). These results show that cyclin D1 did not affect the mitochondrial respiration rate in MM cells. However, the NADPH/NADP+ ratio decreased in cyclin D1-expressing cells (Figure 3B) suggesting increased NOX activity as the availability of its main substrate NADPH was unlimited, in contrast to myeloid cells [27]. The treatment of 8226-derived clones with the pan-NOX inhibitor VAS3947 significantly decreased ROS production in cyclin D1-expressing clones (Figure 3C), confirming the involvement of NOX in cyclin D1-mediated ROS generation.

Cells regulate their intracellular ROS content by balancing ROS production and scavenging systems. We next tested whether the expression of cyclin D1 modifies the expression of detoxifying enzymes. We performed semi-quantitative RT-PCR experiments to analyze the expression of 28 major detoxifying enzymes (Supplementary Table 1). We observed no significant (0.5 < RQ < 2) differences between cyclin D1-expressing and non-expressing clones (Figure 3C). Thus, cyclin D1 disrupts the cellular redox balance by increasing ROS production without modifying the expression of scavenging enzymes encoding genes.

### Inhibition of ROS production reverses the cyclin D1-mediated phenotype

**Figure 4:**
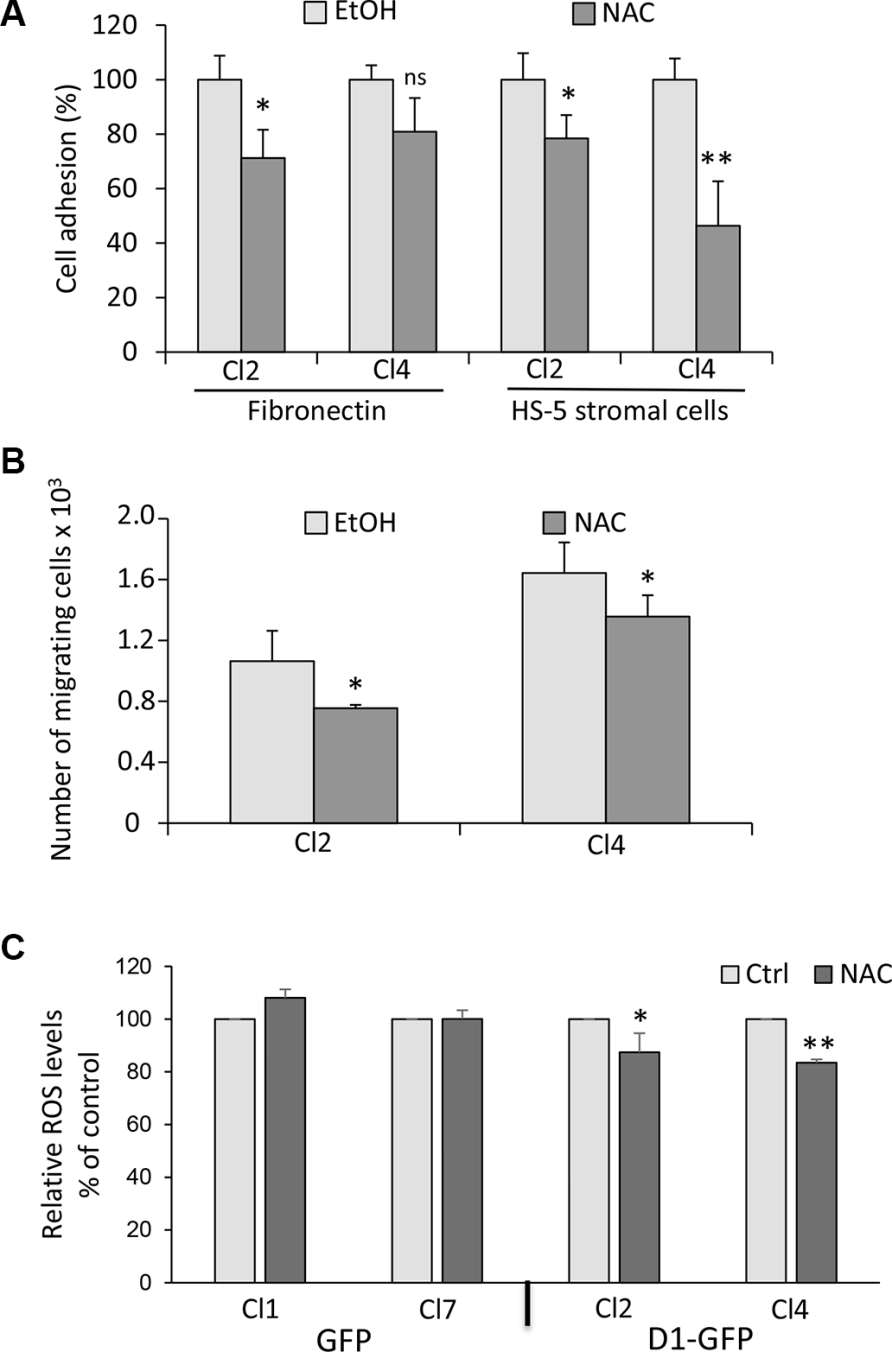
The cyclin D1-induced phenotype is reversed by ROS scavenger (**A**) Cyclin D1-expressing clones (Cl2 and Cl4) were treated with 1 mM NAC or vehicle (EtOH) overnight and tested for adhesion on fibronectin or HS-5 cells as already described. ns, not significant. (**B**) Cl2 and Cl4 were treated as in (A) and tested for chemotaxis as already described. (**C**) GFP− (Cl1 and Cl7) and D1-GFP-expressing cells (Cl2 and Cl4) were treated with 1 mM NAC or vehicle (EtOH) overnight and stained with CellROX to detect relative ROS levels (% of control) in GFP^+^ cells by flow cytometry as already described. At least 2 × 10^4^ events were gated for each clone. The experiment was carried out four times; mean ± SEM are shown on the graph. *p < 0.05; **p < 0.01.

Redox homeostasis modulates myeloma cell drug sensitivity [28–30]. We tested whether the redox state could control adhesion/migration properties of MM cells since the interaction of MM cells with their microenvironment also regulates their responses to drugs. We used *N*-acetylcysteine (NAC) to inhibit ROS production. Cyclin D1-expressing clones were pretreated with NAC before being assayed for adhesion on fibronectin or HS-5 stromal cells (Figure 4A), or for migration (Figure 4B). We observed that NAC induced a decrease in the number of both adherent and migrating cells. We did not observe these effects in GFP-expressing cells (Supplementary Figure 4A, 4B). NAC treatment decreased ROS production in cyclin D1-expressing clones (Figure 4C), suggesting that the redox stress imposed by cyclin D1 is responsible for enhanced adhesion and migration properties.

### Cyclin D1 activates the ERK 1/2 pathway

**Figure 5:**
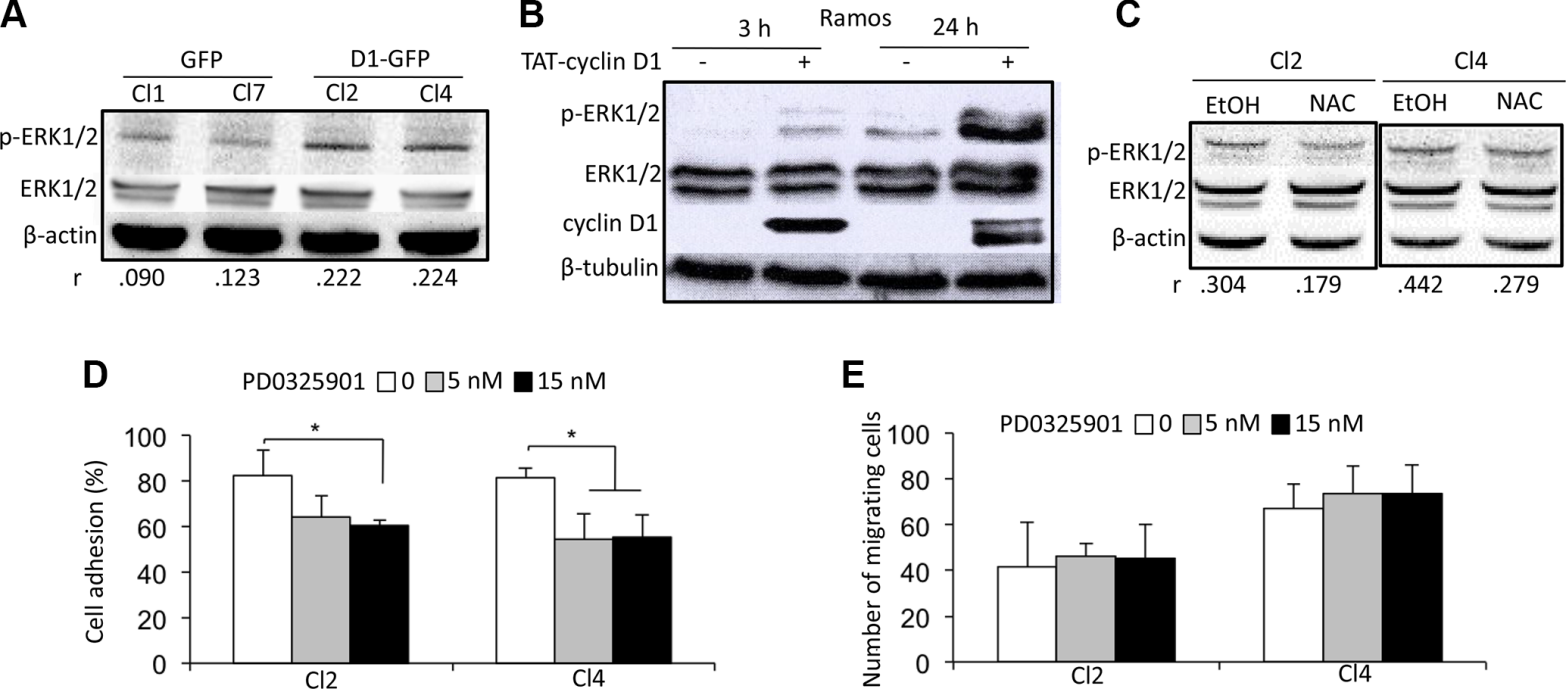
A cyclin D1/ROS/ERK1/2 axis regulates cell adhesion (**A**) Whole-cell protein extracts were obtained from cultured GFP and D1-GFP clones and separated by SDS-PAGE. The proteins were blotted and analyzed using the indicated Abs. An anti-β-actin Ab was used as a loading control. Densitometric analyses were performed on the images captured with the ChemiDoc™ XRS+ molecular imager and analyzed using Image Lab™ software (Bio-Rad). The respective p-ERK1/2/ERK1/2 ratios of GFP- and D1-GFP-expressing clones are indicated under their respective lanes. (**B**) TAT-cyclin D1 fusion protein was produced in bacteria, purified, and directly added to the Ramos cell culture medium (or 0.9% NaCl as a control) as previously described [15]. The cells were harvested 3 or 24 h later for western blot analysis using the indicated Abs. Anti-β-tubulin Ab was used as a loading control. (**C**) D1-GFP-expressing clones (Cl2 and Cl4) were treated with 1 mM NAC for 24 h (or vehicle as a control) and harvested for protein purification and analysis after SDS-PAGE and immunoblotting as before with the indicated Abs. An anti-β-actin Ab was used as a loading control. (**D, E**) D1-GFP-expressing Cl2 and Cl4 were treated with 5 or 15 nM PD0325901 for 24 h, then assayed for cell adhesion on HS-5 stromal cells (D) and chemotaxism (E) as already described.

We next studied which signaling pathways were activated following cyclin D1 expression and ROS generation. Extracellular signal-regulated kinase (ERK)1/2 (or p44/42 mitogen-activated protein kinase) and p70S6 kinase (S6K) phosphorylation was higher in cyclin D1-expressing cells (Figure 5A and Supplementary Figure 5A, upper panel). In contrast, AKT, p38 mitogen-activated protein kinase (MAPK), and STAT3/5 were not activated (data not shown). These results demonstrate that cyclin D1 specifically activates the ERK1/2 and S6K signaling pathways. We used a previously described model of transient expression by transduction of a TAT-cyclin D1 fusion protein in B cells to further confirm this observation [26]. TAT-cyclin D1 protein was rapidly transduced into Ramos cells, and detected as soon as 3 h after the addition of the protein in the culture medium (Figure 5B). After 24 h, TAT-cyclin D1 began to be degraded by the proteasome pathway and was no longer detected after 48 h (data not shown, and ref. 26). Cyclin D1 triggered the phosphorylation of ERK1/2 (Figure 5B) and S6K (Supplementary Figure 5B) after the addition of the fusion protein in the culture medium, consistent with our observations in cyclin D1-expressing MM cells. The treatment of cyclin D1-expressing cells with NAC inhibited the phosphorylation of ERK1/2 proteins and the activation of the pathway (Figure 5C). In contrast, S6K phosphorylation was not affected by NAC treatment (Supplementary Figure 5A, lower panel). Thus, the redox state resulting from the presence of cyclin D1 is necessary for ERK1/2 activation.

We next used rapamycin and PD0325901 to inhibit S6K and ERK1/2 activation, respectively (Supplementary Figure 6A, 6B) to assess the role of their activation on cell adhesion and migration. The inhibition of S6K phosphorylation did not modify the capacity of cyclin D1-expressing cells to adhere to stromal cells or to migrate (Supplementary Figure 6C, 6D). In contrast, the inhibition of ERK1/2 phosphorylation decreased the adhesion of cyclin D1-expressing cells to stromal cells without perturbing their migration (Figure 5D, 5E). We concluded that the redox state imposed by cyclin D1 expression controls cell adhesion in an ERK1/2-dependent, and cell migration in an ERK1/2-independent, manner.

## DISCUSSION

We report here the characterization of new biological functions of cyclin D1 in myeloma cells. We found that cyclin D1 expression generated oxidative stress *via* ROS production which increased cell migration and adhesion, the latter through the activation of the ERK1/2 pathway. To our knowledge, this cyclin D1/ROS/ERK1/2 axis has not been described previously and is particularly relevant for the group of MM patients whose tumor cells express cyclin D1 (CD1/2).

MM is characterized by the development of plasma tumor cells that dynamically and bidirectionnally interact with their bone marrow microenvironment. The genetic and phenotypic heterogeneity of MM may consequently operate at the level of tumor cell/tumor microenvironment interactions [31, 32]. The relationship between the types of soluble and/or physical factors and the molecular groups of MM are largely unknown, except for a high level of β7 integrin associated with the molecular group of MM patients expressing the MAF transcription factor [32]. We report here that cyclin D1 expression increases the expression of ICAM1, and the synthesis of pro-inflammatory chemokines IL8, IP10, and RANTES, all able to alter the tumor microenvironment. Thus, the interaction of MM cells with their tumor microenvironment largely depends on their genetic background.

Several studies have reported that cyclin D1 controls cell adhesion and migration of various cell types and tumors and, consequently, their metastatic potential [8–12, 33]. In mantle cell lymphoma cell lines and primary cells (tumor cells harboring the t(11;14)(q13;q32) and expressing high levels of cyclin D1), sorafenib inhibits cell migration by interfering with B-cell receptor signaling and cyclin D1 translation [34]. In the cell model we have developed, cyclin D1 expression results in increased adhesion of MM cells on fibronectin and stromal HS-5 cells. In sharp contrast with solid tumors for which adhesion and migration are inversely coordinated, cyclin D1 also confers greater migratory capacity. We found that ICAM1 was over-synthesized and the chemokines IP10, RANTES and IL8 overproduced in cyclin D1-expressing cells. How cyclin D1 regulates chemokine and interleukin production is an open question. Our transcriptomic data showed that cyclin D1 expression did not modify *CXCL10*, *CCL5*, or *CXCL8* genes transcription [7 and data not shown], suggesting that cyclin D1 may act at a post-transcriptional level. In mouse fibroblasts, cyclin D1 is localized to trans-Golgi and exocyst-rich regions, binds small GTPases RalA and B, and the exocyst protein SEC6, all involved in the regulation of exocytosis [35]. As plasma cells possess highly developed secretory machinery, this is a reasonable hypothesis.

We have previously shown that IP10, RANTES, and IL8 are secreted by MM cells and that IP10 and RANTES are over-produced after genotoxic stresses, resulting in a senescence-associated secretory phenotype which allows MM cells to migrate [36]. The common response of MM cells exposed to X-irradiation, DNA damaging agents, or constitutive cyclin D1 expression is ROS generation [36 and this study]. MM cells exhibit higher intrinsic oxidative stress than normal cells, as do most cancer cells, and are adapted to this redox status. This is achieved by the up-regulation of thioredoxin (TRX1) and thioredoxin reductase (TRXR1), which are ROS scavengers and regulators of redox enzymes [37]. The proteasome inhibitors bortezomib and carfilzomib, which are widely used in the clinic, induce apoptosis by increasing intracellular ROS levels and generating an oxidative stress [38, 39]. This is considered to be a “side effect” of the endoplasmic reticulum (ER) stress generated by the accumulation of unfolded proteins and the stimulation of the unfolded protein response (UPR) pathway. We previously showed that cyclin D1 expression in MM cells activates the UPR pathway and favors cell death through the protein kinase R-like endoplasmic reticulum kinase (or PERK)/activating transcription factor 4 (or ATF4)/CCAAT enhancer binding homologous protein (or CHOP) axis [7]. Thus, cyclin D1 may indirectly impair redox homeostasis through an UPR-mediated ER stress in MM cells. However, bortezomib and carfilzomib act also directly *via* the transcriptional repression of mitochondrial thioredoxin reductace (TXNRD2), a ROS detoxifying enzyme that maintains the intracellular redox status [40]. We found no transcriptional modification of detoxifying enzymes associated with cyclin D1 expression. In contrast, we showed that the generation of ROS, that perturbed the redox balance, was due to the activity of NOX or dual oxidases (DUOX) and their associated subunits.

NOX/DUOX proteins belong to a family of flavoproteins that transports electron across biological membranes and generates ROS [41]. The most common NOX isoform found in B cells is NOX2 (gp91^phox^/cytochrome b_558_). NOX2 produces ROS in B cells and participates into lymphoma and leukemia cells death [42, 43]. How NOX and their regulatory subunits are regulated in MM cells and how cyclin D1 controls the expression, assembly and activity of the various NOX complexes, merits further investigations. NOX links NADPH metabolism and migration in myeloid cells transformed by oncogenic tyrosine kinases [27]. Moreover, oncogenes with tyrosine kinase activity, such as BCR/ABL, Flt3-ITD, and c-Kit, alter the redox homeostasis in leukemic cells contributing to proliferative and anti-apoptotic effects [27]. Cyclin D1 is an oncogene that alters genomic stability, survival, growth and adhesion/motility [5], all which are potentially controlled by ROS production.

We show that ROS signals through the activation of the ERK1/2 pathway in MM cells in agreement with a previous report [44]. Moreover, we show that the down-regulation of ERK1/2 phosphorylation, using a specific inhibitor, decreases the adhesion of MM cells. The Ras/Raf/MEK/ERK cascade plays a central role in the regulation of MM cell adhesion, migration, and homing [45]; this is in good agreement with our data.

Myeloma cell death can be achieved through the generation of ROS that follows ER stress and UPR activation [46]. Elevating the level of intracellular ROS in myeloma cells synergizes with various compounds to inhibit MM cell growth or/and trigger apoptosis [47, 48]. Manipulating redox parameters could improve the therapeutic response of MM patients, especially for those belonging to the cyclin D1-expressing group.

## MATERIALS AND METHODS

### Chemicals

Bortezomib (or PS-341), carfilzomib (or PR-171), pomalidomide and PD0325901 (a selective inhibitor of mitogenic extracellular kinase) were purchased from Selleckchem. VAS3947, a pan-NOX inhibitor was purchased from Calbiochem. Rapamycin and NAC were purchased from Sigma-Aldrich. All chemicals were dissolved in dimethyl-sulfoxide (DMSO), except NAC which was dissolved in ethanol (EtOH). For controls, the vehicle was added to the same final concentration.

### Cell lines and cell culture, and transduction of TAT-cyclin D1 fusion protein

8226-derived clones were maintained in RPMI 1640 medium (Lonza) supplemented with 2 mM L-glutamine (Lonza), 10% FCS (PAA Laboratories) and antibiotics (Lonza). 8226 cells were purchased from DSMZ (ACC-402). The 8226 GFP- and D1-GFP-expressing clones were obtained after stable transfection of the corresponding expression plasmids, selection with Geneticin, cloning by limiting dilution, and analysis by flow cytometry on FL1-fluorescence. This strategy has been previously described in detail [7]. The transduction method of TAT-cyclin D1 protein in B cells has been previously described in detail [26].

### Detection of intracellular ROS

Intracellular ROS were detected using the oxidation-sensitive fluorescent probe CellROX^®^ Deep Red reagent (Life Technologies) according to the manufacturer's instructions.

### Quantitative determination of the NADP/NADPH ratio

The determination of the NADPH/NADP^+^ ratio (from 10^6^ cells) was performed using the NADP^+^/NADPH Assay Kit (ab176724, Abcam). This colorimetric assay was carried out according to the manufacturer's instructions. The optical density was read at 450 nm with the Victor™ X4 plate-reader (Perkin Elmer). The protein concentration was determined for each sample and the values represented as picomoles NADPH and NADP+ per μg of lysate.

### Adhesion and chemotaxis assays

96-well plates were coated overnight at room temperature with fibronectin (10 μg/ml in PBS, 100 μl/well), and extensively washed in PBS. The human stromal cell line HS-5, obtained from the ATCC (CRL-11882), was maintained in Dulbecco's modified Eagle's medium containing antibiotics, L-glutamine and 10% FCS. HS-5 cells (2 × 10^4^ cells) were seeded in a volume of 100 μl in each well of 96-well plates and cultured for two days until they reached confluence. Cell adhesion was assessed with the Vybrant™ Cell Adhesion Assay Kit (V-13181, Molecular Probes). Briefly, MM cells were harvested, washed, and labeled with calcein acetoxymethyl ester (calcein-AM, 1 mM) for 30 min and stained cells (5 × 10^4^/well) were seeded on fibronectin- or HS-5-coated plates and incubated at 37°C for 3 or 24 h. After extensive washing to remove non-adherent calcein-labeled cells, the plates were read using the Victor 4 (Perkin Elmer) to measure the fluorescence of adhering cells (Ex 494 nm/Em 517 nm). Bovine serum albumin (BSA)–coated wells served as negative controls, and poly-L-lysine–coated wells served as positive controls.

For the cell migration or chemotaxis assay, cultured MM cells (5 × 10^5^ cells per insert) were washed and suspended in RPMI 1640 medium containing 0.5% BSA. They were added in the top chambers of transwell inserts (Millicell^®^ Hanging Cell Culture Inserts 8 μm PET, Millipore). The filters were transferred to wells containing medium with 10% FCS as chemoattractant. As a control for the specificity of the assay, FCS-free medium was added in the lower chamber. The plates were then incubated for 4 h at 37°C and cells migrating to the lower chambers were counted by flow cytometry (300 s under constant flow).

### Indirect immunofluorescence and confocal microscopy analysis

Cells (10^5^ cells per spot) were cytospun on Superfrost glass slides, at 500 × g for 3 min, then fixed in 4% paraformaldehyde (PFA), and permeabilized by incubation with 0.5% Triton-X100 (v/v) for 5 min. The slides were then stained with a rhodamine-stained phalloidin probe (Molecular Probes) for selectively visualizing F-actin, and DAPI (4′,6-diamidino-2-phenylindole dihydrochloride, Molecular Probes) for nuclear counterstaining. The slides were mounted, and analyzed with a Fluoview FV 1000 confocal microscope and Fluoview Viewer software (Olympus).

### Apoptosis assessment

MM cells were treated with 50−100 nM carfilzomib or/and 1 μM pomalidomide and stained with PE-conjugated anti-APO2.7 antibody (Ab) and analyzed by flow cytometry. For co-culture experiments, HS-5 and MM cells were stained with APO2.7-PE- and CD10-APC-conjugated antibodies (Miltenyi). Only CD10-negative cells corresponding to myeloma cells were analyzed.

### Detection of produced cytokines and chemokines

The Proteome Profiler™ Human Cytokine Array Kit, Panel A was used for the determination of the relative levels of 36 selected cytokines and chemokines. We directly analyzed the supernatant of cultured GFP and D1-GFP clones as recommended by the manufacturer (R & D systems, Minneapolis, MN), 72 h after cell seeding.

### Western blotting

Western blotting was performed as previously described in Bustany *et al*. [7]. We used primary Abs against ERK1/2 (#9102), pThr202/Tyr204-ERK1/2 (#9101), S6K (#9202), and pThr389-S6K (#9205) from Cell Signaling Tech. The Abs against cyclin D1 (sc-718), β-actin (sc-47778), and β-tubulin (sc-9104) were purchased from Santa Cruz Biotech. The secondary Abs were goat anti-rabbit or anti-mouse peroxidase-conjugated IgGs (Abcam). For densitometric analyses, images were captured with a ChemiDoc™ XRS+ molecular imager and analyzed using Image Lab™ software (Bio-Rad). The background of each image was subtracted from the bands of interest, then the densities of each protein were normalized against the density of β-actin used as a control housekeeping protein. The ratios of the normalized values: p-ERK1/2/ERK1/2 are indicated under the corresponding blots.

### Semi-quantitative RT-PCR analyses

Cultured cells were washed once in PBS and total RNA was isolated from pelleted cells using the Trizol reagent according to the manufacturer's instructions. The RNA was reverse-transcribed using the SuperScript^®^ VILO cDNA Synthesis Kit (Invitrogen). PCR primers (Supplementary Table 1) were designed using ProbeFinder software (Roche Applied Software). cDNAs, primers (0.25 μM), and LightCycler^®^ TaqMan^®^ Master mix were mixed in a final volume of 10 μl and PCR-amplified in a LightCycler^®^ 480 Instrument II (Roche). The amplification parameters were the following: an initial denaturation step at 95°C for 5 min, 45 cycles at 95°C for 10 s, and 60°C for 30 s, and a final cooling step at 40°C for 30 s. The *GAPDH* gene was used as an internal gene for normalization. Each reaction condition was performed in triplicate. Relative gene expression was evaluated by the 2^−ΔΔCt^ method.

### Statistical analysis

The Student's *t*-test was used to determine the significance of differences between two experimental groups. Data were analyzed by two-tailed tests, with *p* < 0.05 (*) considered to be significant.

## Supplementary Materials Figures and Tables


