# Supplementary material for: Cyclin D1 unbalances the redox status controlling cell adhesion, migration, and drug resistance in myeloma cells
Source: Oncotarget. 2016 Jun 7;7(29):45214–24. doi: 10.18632/oncotarget.9901 (PMC5216717; doi:10.18632/oncotarget.9901)
Supplement: Supplementary file 1 [file oncotarget-07-45214-s001.pdf]

Cyclin D1 unbalances the redox status controlling cell adhesion, migration, and drug resistance in myeloma cells

Supplementary Materials

SUPPLEMENTARY METHODS

Cellular oxygen consumption rate (OCR) measurements were obtained using a Seahorse XF96 Flux analyzer (Seahorse Bioscience). Experiments were performed according to the manufacturer's instructions. Briefly, cell lines were seeded with poly-D-lysine in XF96 cell culture plates at 10^5 cells/well. The XF96 sensor cartridges were hydrated overnight with 200 µl Calibrant at pH7.4 and stored at 37°C without CO_2 for 24 h. The culture medium was replaced with XF BASE MEDIUM supplemented with glutamine (2 mM) and lacking bicarbonate (pH7.4). The cells were then incubated for 1 h at 37°C without CO_2 and measurements were performed.

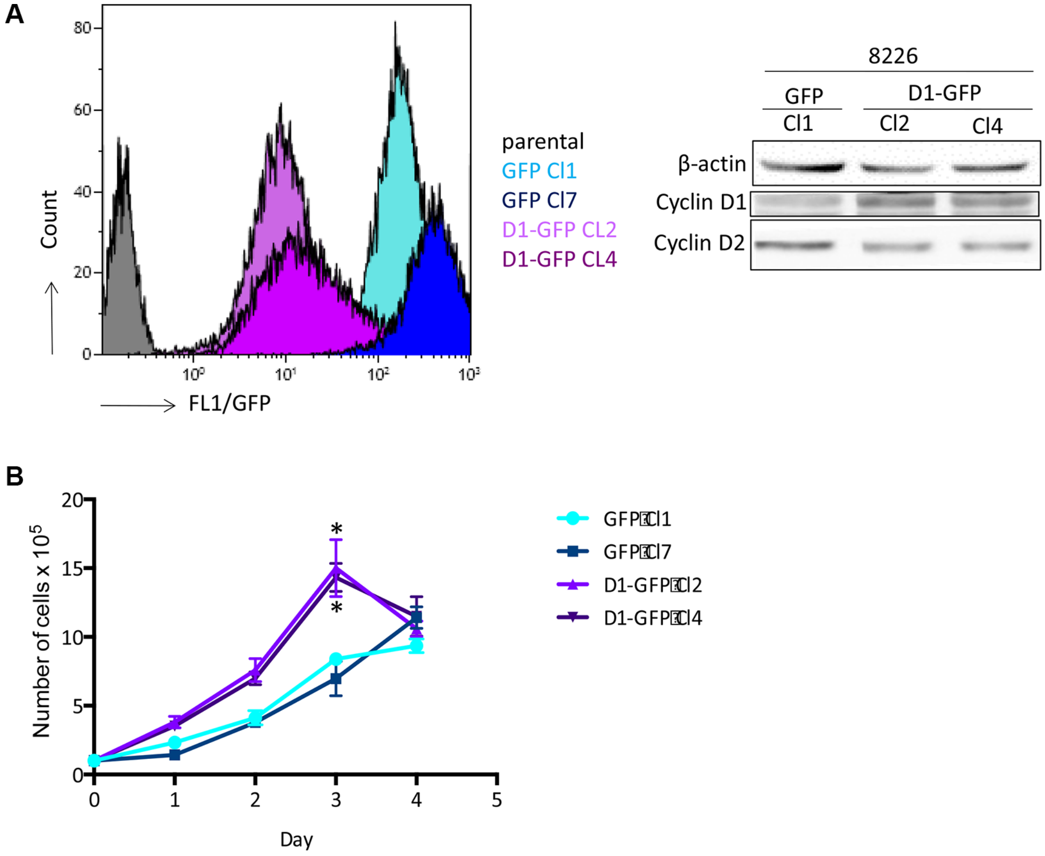

Supplementary Figure S1: Characterization of GFP- and D1-GFP-expressing clones by flow cytometry and immunoblotting. (**A**) 8226 parental cells were transfected with expression plasmids coding for GFP or cyclin D1-GFP proteins, and the resulting clones selected by antibiotic resistance. Two clones from each series (Cl1 and Cl7 for GFP-expressing cells and Cl2 and Cl4 for D1-GFP expressing cells) and parental cells were analyzed by flow cytometry for GFP staining (FL1). At least 2×10^4 events were gated for each series. Representative profiles are shown on the graph. Proteins were purified from GFP- and D1-GFP-expressing cultured cells, separated on SDS-PAGE, blotted onto nitrocellulose membranes, and incubated with the indicated Abs. An anti β-actin Ab served as a loading and transfer control. (**B**) 8226 GFP- (Cl1 and Cl7) and D1-GFP- (Cl2 and Cl4) expressing cells were seeded in 24-well plates at the density of 10^5 cells/ml and cultured in complete medium. The cells were counted every day during a four-day period. The number of living cells was plotted as a function of time (day in culture). The experiment was carried out three times with triplicate samples. $*p < 0.05$ with the *t*-test.

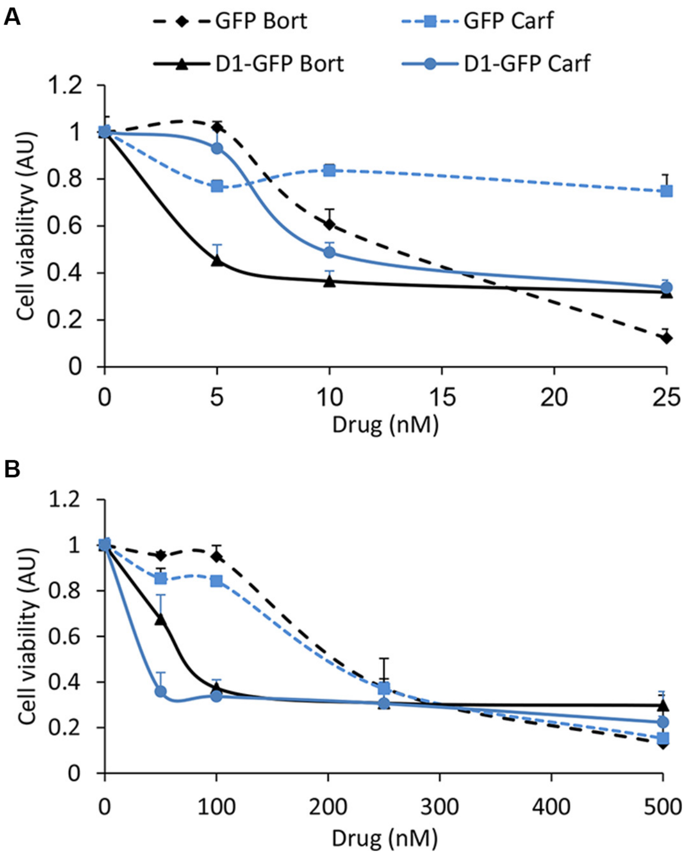

Supplementary Figure S2: Effects of bortezomib and carfilzomib on cell viability assessed by a MTS assay. 5×10^5 GFP-expressing cells (Cl7, black diamonds and blue squares, dotted lines) and D1-GFP-expressing cells (Cl4, black triangles and blue circles, solid lines) were treated for 24 h (**A**) or 1 h (**B**) with bortezomib (black lines) or carfilzomib (blue lines) and cell viability was assessed by a MTS assay (here the CellTiter 96® AQ_{ueous} One Solution Cell Proliferation Assay, Promega) according to the manufacturer's recommendations. The absorbance (OD at 490 nm) of each clone treated with the drug is expressed relative to that of the corresponding clone treated with vehicle (defined as 1 in arbitrary units, AU). For each set of culture conditions, the mean of triplicate ratios is indicated on the graph, together with SD.

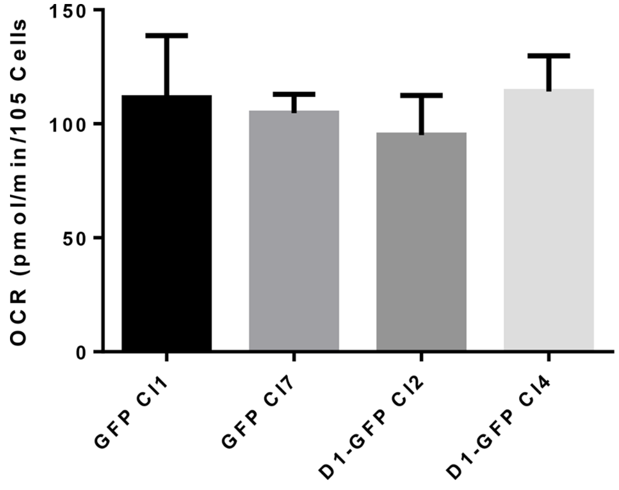

Supplementary Figure S3: Effects of cyclin D1 expression on the cellular oxygen consumption rate. OCRs (pmol/min/10^5 cells) were measured by a Seahorse Analyzer in GFP- (Cl1 and Cl7) and D1-GFP-expressing cells (Cl2 and Cl4). Basal measurements were normalized for cell concentration and the mean ± SEM from four independent experiments are plotted.

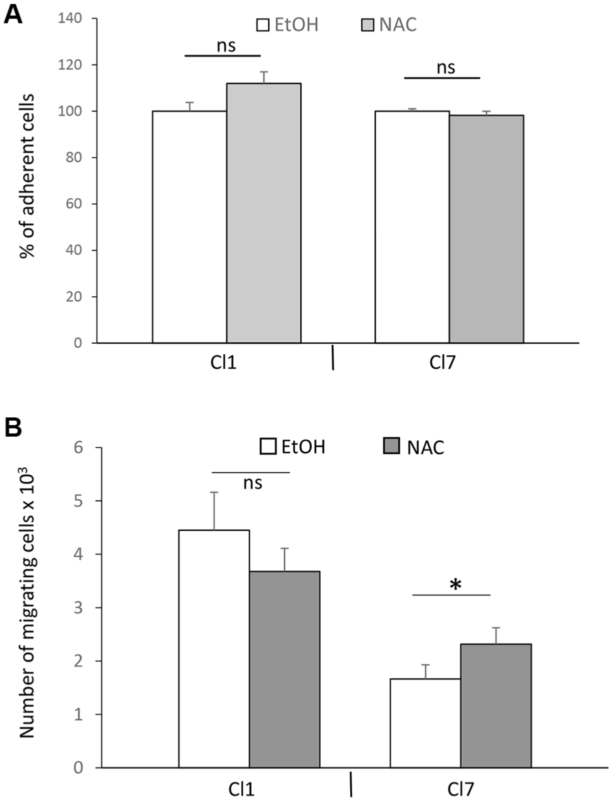

Supplementary Figure S4: NAC treatment has no impact on adhesion and minor effect on migration. GFP-expressing clones (Cl1 and Cl7) were treated with 1 mM NAC for 24 h and adhesion (**A**) and migration (**B**) assayed with the described protocols. ns, not significant; $*p < 0.05$ by the *t*-test.

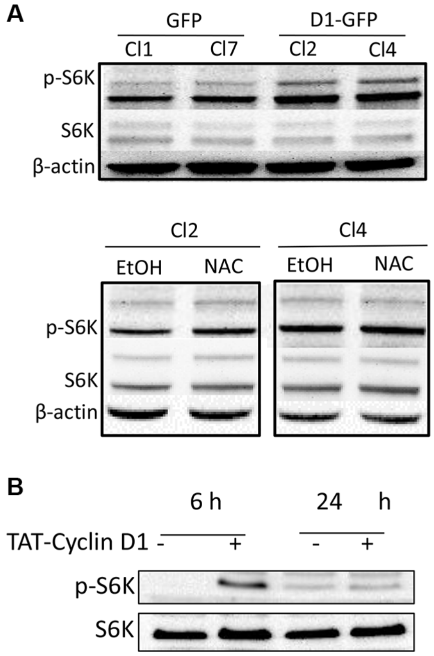

Supplementary Figure S5: Cyclin D1 expression is accompanied by the activation of the S6K signaling pathway. (**A**) *Upper panel*. Whole-cell protein extracts were obtained from cultured GFP and D1-GFP clones and separated by SDS-PAGE. The proteins were blotted and analyzed using the indicated Abs. An anti-β-actin Ab was used as a loading control. *Lower panel*. D1-GFP-expressing clones (Cl2 and Cl4) were treated with 1 mM NAC for 24 h (or vehicle as control) and harvested for protein purification and analysis after SDS-PAGE and immunoblotting as before with the indicated Abs. (**B**) TAT-cyclin D1 fusion protein was produced in bacteria, purified and directly added to the Ramos cell culture medium (or 0.9% NaCl as control) as previously described (Tchakarska *et al*., Cancer Res. 2011;71,1690). The cells were harvested 3 or 24 h later for western blot analysis using the indicated Abs.

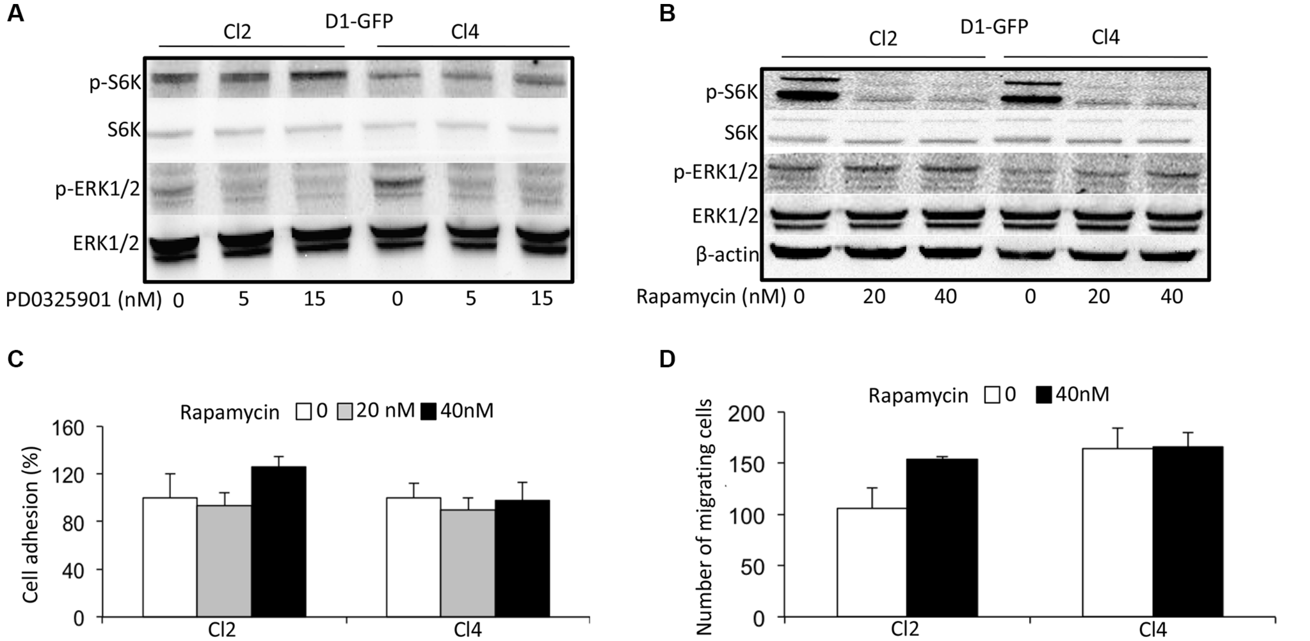

Supplementary Figure S6: The inhibition of S6K by rapamycin has no effects on MM cell adhesion and migration. Cyclin D1-expressing cells (D1-GFP Cl2 and Cl4) were treated with 5−15 nM PD0325901 or 20−40 nM rapamycin, or vehicle (0) for 24 h and the cells were analyzed by western blotting for S6K and ERK1/2 expression and activation (**A**) and (**B**) , cell adhesion on HS-5 stromal cells (**C**) and chemotaxism (**D**) .

Supplementary Table S1: Sequences of the primers used for RT-PCR

Gene symbol	Gene product	Primer sequences	
		Forward	Reverse
CAT	Catalase	cgcagttcggttctccac	gggtcccgaactgtgtca
GAPDH	Glyceraldehyde dehydrogenase	agccacatcgctcagacac	gcccaatacgaccaaatcc
GLRX(1 & 2)	Glutaredoxin 1 (thioltransferase)	ggcttcggaatttgtcgat	tgcatccgccratacaatctt
GLRX2 (1)	Glutaredoxin 2, variant 1	gtggcactcgctggaatc	cgtcgctaaattctccaaagat
GLRX2 (2)	Glutaredoxin 2, variant 2	gctggtttggagcaggag	ccaaagatgatgatgtattgctct
GLRX3	Glutaredoxin 3	tctcaagaaccacgctgt	tgagaagatatcaaaactgctaaactg
GLRX5	Glutaredoxin 5	gtgataactggggcgttgtt	actcaggcatgcacagca
GPX1 (1)	Glutathione peroxidase 1, variant 1	caaccagtttgggcatcag	gttcacctcgcacttctcg
GPX1 (2)	Glutathione peroxidase 1, variant 2	cccttgtttgtggttagaacg	gagagaagggcagctagaacc
GPX2	Glutathione peroxidase 2	gtccttggcttcccttgc	tgttcaggatctcctcattctg
GPX3	Glutathione peroxidase 3	cagagatccttcctaccctcaa	ccctttctcaaagagctgga
GPX4 (1,2 & 3)	Glutathione peroxidase 4, variants 1, 2 & 3	tacggacccatggaggag	ccacacacttgtggagctagaa
GPX5 (1 & 2)	Glutathione peroxidase 5, variants 1 & 2	tgctttgtgcaaacaagtcc	ggtgcctttctcgtctttgt
GPX6	Glutathione peroxidase 6	aggcttggcagctcagtatc	gccaacacaatgacaccaaa
GPX7	Glutathione peroxidase 7	ccatcctgccttcaagtacc	ttccatcggggctactagg

GSR	Glutathione reductase	tgccagcttaggaataaccag	cctgcaccaacaatgacg
PRDX1 (1,2 & 3)	Peroxiredoxin 1, variants 1,2 & 3	cactgacaaacatggggaagt	tttgctcttttggacatcagg
PRDX2 (1)	Peroxiredoxin 2, variant 1	gccttccagtacacagacgag	gttgggcttaatcgtgtcact
PRDX2 (3)	Peroxiredoxin 2, variant 3	gcaactcagatgcaactctatctact	tgaactggagtttccatcttcat
PRDX3 (1 & 2)	Peroxiredoxin 3, variants 1 & 2	ctggacaccggattctccta	gggtgatctactgatttaccttctg
PRDX4	Peroxiredoxin 4	gcacctaagcaaagcgaaga	aaattctccatcgatcacagc
PRDX5 (1 & 3)	Peroxiredoxin 5, variants 1 & 3	tcctggctgatcccactg	atgccatcctgtaccaccat
PRDX5 (2)	Peroxiredoxin 5, variant 2	cacccctggatgttccaa	ggacaccagcgaatcatctagt
PRDX6	Peroxiredoxin 6	caatagacagtgttgaggaccatc	tttctgtgggctcttcacaa
SOD1	Superoxide dismutase 1	gcatcatcaatttcgagcag	caggccttcagtcagtcctt
SOD2	Superoxide dismutase 2	tccactgcaaggaacaacag	taagcgtgctcccacacat
SOD3	Superoxide dismutase 3	ctctcttttcaggagagaaagctc	aacacagtagcgccagcat
TXN	Thioredoxin	ttacagccgctcgtcaga	ggcttcctgaaaagcagtctt
TXN2	Thioredoxin 2	gagacaccagtggttgtgga	gcttggccaccatcttctc
